# Potential antipsychotic action of the selective agonist of adenosine A1 receptors, 5′-Cl-5′-deoxy-ENBA, in amphetamine and MK-801 rat models

**DOI:** 10.1007/s43440-020-00093-3

**Published:** 2020-03-26

**Authors:** Krystyna Ossowska, Barbara Kosmowska, Jadwiga Wardas

**Affiliations:** Department of Neuropsychopharmacology, Maj Institute of Pharmacology, Polish Academy of Sciences, 31-343 Kraków, Poland

**Keywords:** Adenosine A1 receptors, Amphetamine, MK-801, Hyperlocomotion, Rat

## Abstract

**Background:**

Disturbances of dopaminergic and glutamatergic transmissions have been suggested to be involved in the pathomechanisms underlying psychotic symptoms of schizophrenia. In line with this concept, hyperlocomotion induced by the dopaminomimetic amphetamine and the uncompetitive antagonist of NMDA receptors MK-801 (dizocilpine) in rodents is a generally established model for screening of new potential antipsychotic drugs. Since recent studies have indicated that receptors for adenosine may be targets for antipsychotic therapy, the aim of the present study was to investigate an influence of 5′-Cl-5′-deoxy-ENBA, a potent and selective adenosine A_1_ receptor agonist, on hyperlocomotion induced by amphetamine and MK-801.

**Methods:**

Locomotor activity was measured by Force Plate Actimeters where four force transducers located below the corners of the floor of the cage tracked the animal position on a Cartesian plane at each time point.

**Results:**

Hyperlocomotion induced by either amphetamine (1 mg/kg sc) or MK-801 (0.3 mg/kg ip) was inhibited by 5′-Cl-5′-deoxy-ENBA (0.1 mg/kg ip). The effect of 5′-Cl-5′-deoxy-ENBA on the amphetamine- and MK-801-induced hyperlocomotion was antagonized by the selective antagonist of adenosine A_1_ receptor DPCPX at doses of 1 and 2 mg/kg ip, respectively.

**Conclusion:**

The present study suggests that stimulation of adenosine A_1_ receptors may produce antipsychotic effects.

## Introduction

Schizophrenia is one of the most prevalent mental disorders. It is characterized by an appearance of the so-called “positive” (delusions, hallucinations, disorganized thinking) and “negative” (anhedonia, blunted affect, social withdrawal) symptoms, as well as affective disorders (depression or mania), and cognitive disturbances [1]. Although some progress has been made both in understanding of schizophrenia pathomechanisms and pharmacotherapy during recent decades, this disease is still incurable and its symptoms cannot be successfully controlled, yet.

Two main hypotheses of schizophrenia pathomechanisms are currently accepted that claim that hyperfunction of dopaminergic and hypofunction of glutamatergic transmission underlie symptoms of this disease. In line with these concepts, amphetamines, which enhance the release of dopamine in the striatum, can exacerbate psychotic symptoms in schizophrenia, and produce positive symptoms in healthy subjects, which may develop into primary psychosis [1]. Both the amphetamine- and schizophrenia-induced positive symptoms are alleviated by neuroleptics whose therapeutic potency has been shown to correlate with blockade of D_2_ dopamine receptors [1]. On the other hand, phencyclidine (PCP), ketamine and other NMDA receptor antagonists are known to induce both positive and negative symptoms in healthy humans and may precipitate schizophrenia [1].

Based on the above-mentioned putative mechanisms underlying schizophrenia symptoms, a number of animal (rodent) models have been developed. Among them, hyperlocomotion induced by dopaminomimetics (amphetamines, cocaine) and uncompetitive NMDA receptor antagonists [PCP, ketamine, MK-801 (dizocilpine)], which is inhibited by neuroleptics, seems to model positive symptoms in schizophrenia patients. This behaviour is commonly used to screen of new potential antipsychotic agents [1].

Besides dopamine and glutamate, dysfunctions of other neurotransmitter/neuromodulator systems, e.g. adenosine have been suggested to play a role in the pathomechanisms of schizophrenia [1, 2].

Adenosine, a ubiquitous neuromodulatory nucleoside acts mainly, but not solely, through G-protein-coupled A_1_, A_2A_, A_2B_ and A_3_ receptors [3]. While inhibitory A_1_ and A_3_ adenosine receptors decrease adenylyl cyclase activity and cAMP level, facilitatory A_2A_ and A_2B_ receptors induce an opposite effect, i.e. activate adenylyl cyclase and increase cAMP level [3]. Adenosine receptors have been suggested to be potential therapeutic targets in several central nervous system disorders, e.g. epilepsy, brain ischemia, pain and inflammation, Parkinson’s disease, essential tremor and others [3–5]. It has been hypothesized that dysregulation of adenosine neuromodulation may influence neurodevelopment in schizophrenia and contribute to appearance of its symptoms [1, 2]. In line with this concept, both adenosine A_1_ and A_2A_ receptor agonists have been found to inhibit hyperlocomotion or sensorimotor gating deficits induced by dopaminomimetics and/or NMDA receptor channel blockers in rodents [1, 2]. However, most animal studies have suggested sedative and amnestic effects of adenosine A_1_ agonists which can be disadvantageous in schizophrenia [1]. On the other hand, antagonists of A_1_ and A_2A_ or a non-selective antagonist of adenosine receptors—caffeine—exhibited procognitive properties in memory impairment models [1]. In line with the latter findings, caffeine consumption is higher in schizophrenia patients probably because its use is associated with improved semantic fluency, cognitive speed, working and visual memory, and counteracting the medication-induced sedation [6]. Moreover, agonists of both these receptors induce also some peripheral effects which may limit their therapeutic use in brain diseases [7–9]. As far as adenosine A_1_ receptor agonists are concerned, they induce negative dromo- and chronotropic effects in humans [8], slow down the heart rate, and decrease systolic blood pressure in animals [7, 10].

The aim of the present study was to examine potential antipsychotic properties of 5′-chloro-5′-deoxy-( ±)-ENBA (5′-Cl-5′-deoxy-ENBA), a potent and selective adenosine A_1_ receptor agonist, which binds to this receptor with nanomolar affinity (Ki = 0.20–0.51 nM), which is 2500–20,000 times higher than that for A_2A_, A_2B_ or A_3_ receptors, and penetrates the blood–brain barrier [11–14]. 5′-Cl-5′-deoxy-ENBA has already been found to reduce pain in the formalin test [11] or hyperalgesia and mechanical allodynia in the model of the neuropathic pain in mice [12]. This compound decreased locomotor activity and l-DOPA dyskinesia in mice and rats [5, 13], and the harmaline-induced tremor (a model of essential tremor) in rats [5]. 5′-Cl-5′-deoxy-ENBA appeared better than other agonists of A_1_ adenosine receptors because its pharmacologically active doses were devoid of peripheral side effects, i.e. it did not affect heart rate or systolic blood pressure [12].

Our recent study has indicated that 5′-Cl-5′-deoxy-ENBA administered in rats decreased hyperactivity induced by harmaline [5]. Since harmaline is known to produce psychomotor agitation and hallucinations in humans [15], the above-mentioned effect of 5′-Cl-5′-deoxy-ENBA may suggest its antipsychotic properties. However, since the harmaline-induced hyperactivity in rodents is not an established model of psychoses, in the present study, we examined an antipsychotic potential of 5′-Cl-5′-deoxy-ENBA in two classical models, i.e. the amphetamine- and MK-801-induced increase in locomotor activity in rats.

## Materials and methods

### Animals

The experiments were carried out according to the EU Directive 2010/63/EU for animal experiments and were approved by the Local Ethics Committee at the Institute of Pharmacology (permission no: 1069/2013; annex 1069/2016; 234/2017). All efforts were made to minimize the number and suffering of animals used. Male Wistar Han rats (310–350 g) were kept under a 12/12-h light/dark cycle (the light on from 7 am to 7 pm) with free access to food and water. All experiments were carried out during the light period.

### Drugs

**Fig. 1:**
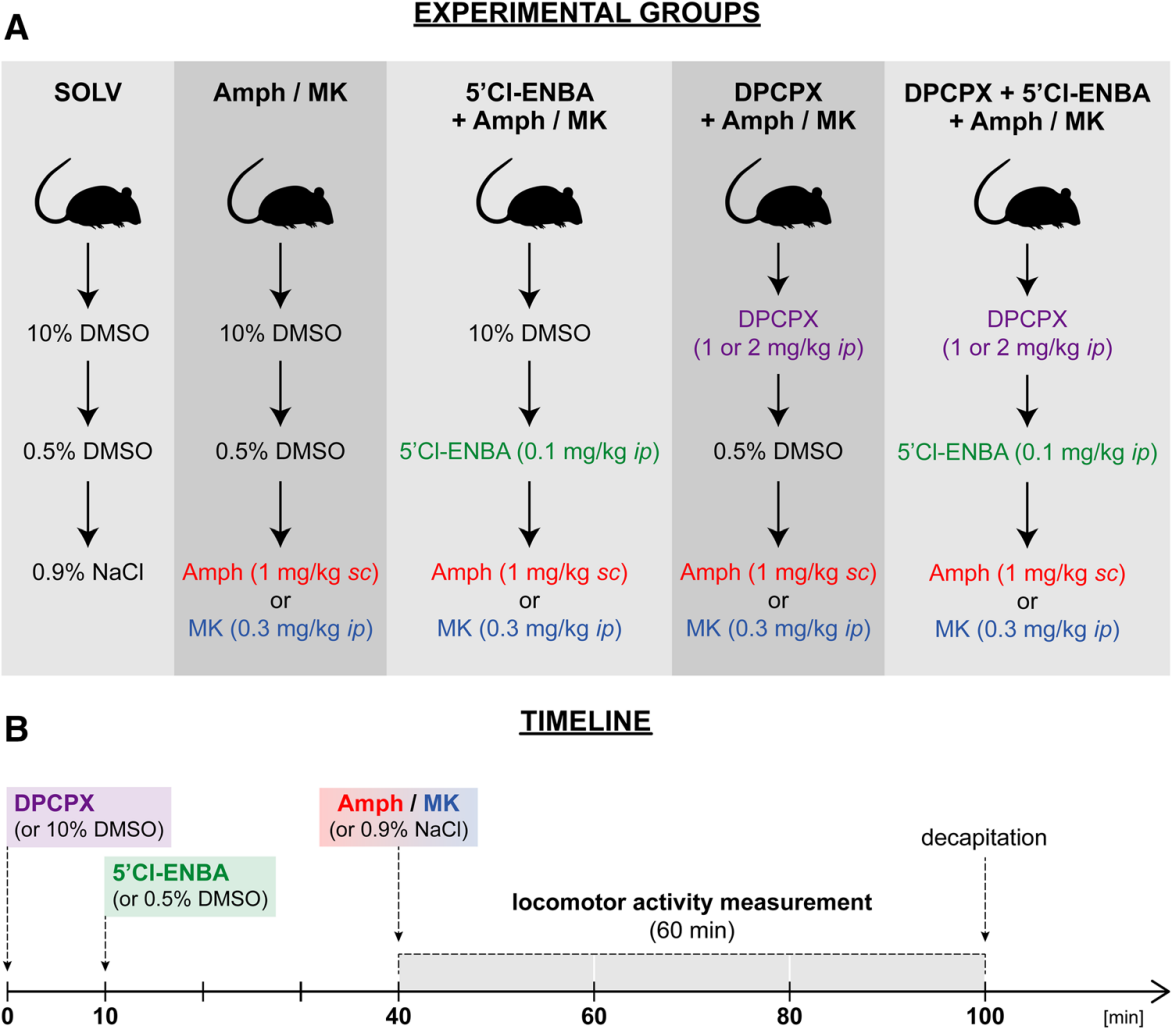
A description of groups of rats and time schedule of the experiment. *5'Cl-ENBA* 5'-Cl-5'-deoxy-ENBA, *Amph* amphetamine, *MK* MK-801 (dizocilpine), *SOLV* solvent

d-Amphetamine hemisulfate salt (Sigma-Aldrich, Saint Louis, USA) and MK-801 (dizocilpine, Tocris Bioscience, Bristol, UK) were dissolved in physiological saline and administered at doses of 1 mg/kg sc, and 0.3 mg/kg ip, respectively. 5′-Chloro-5′-deoxy-N^6^-( ±)-(endo-norborn-2-yl)adenosine (5′-Cl-5′-deoxy-ENBA, Tocris Bioscience, Bristol, UK) was dissolved in 0.5% DMSO in physiological saline and administered at a dose of 0.1 mg/kg ip 30 min before amphetamine or MK-801. 8-Cyclopentyl-1,3-dipropylxanthine, a selective antagonist of adenosine A_1_ receptors (DPCPX, Tocris Bioscience, Bristol, UK) [16] was dissolved in 10% DMSO in physiological saline and administered at doses of 1 or 2 mg/kg ip 10 min before 5′-Cl-5′-deoxy-ENBA (40 min before amphetamine or MK-801). Physiological saline was used as the control for amphetamine and MK-801, 0.5% DMSO for 5′-Cl-5′-deoxy-ENBA and 10% DMSO for DPCPX (Fig. 1).

### Measurement of locomotor activity in force plate actimeters (FPA)

**Fig. 2:**
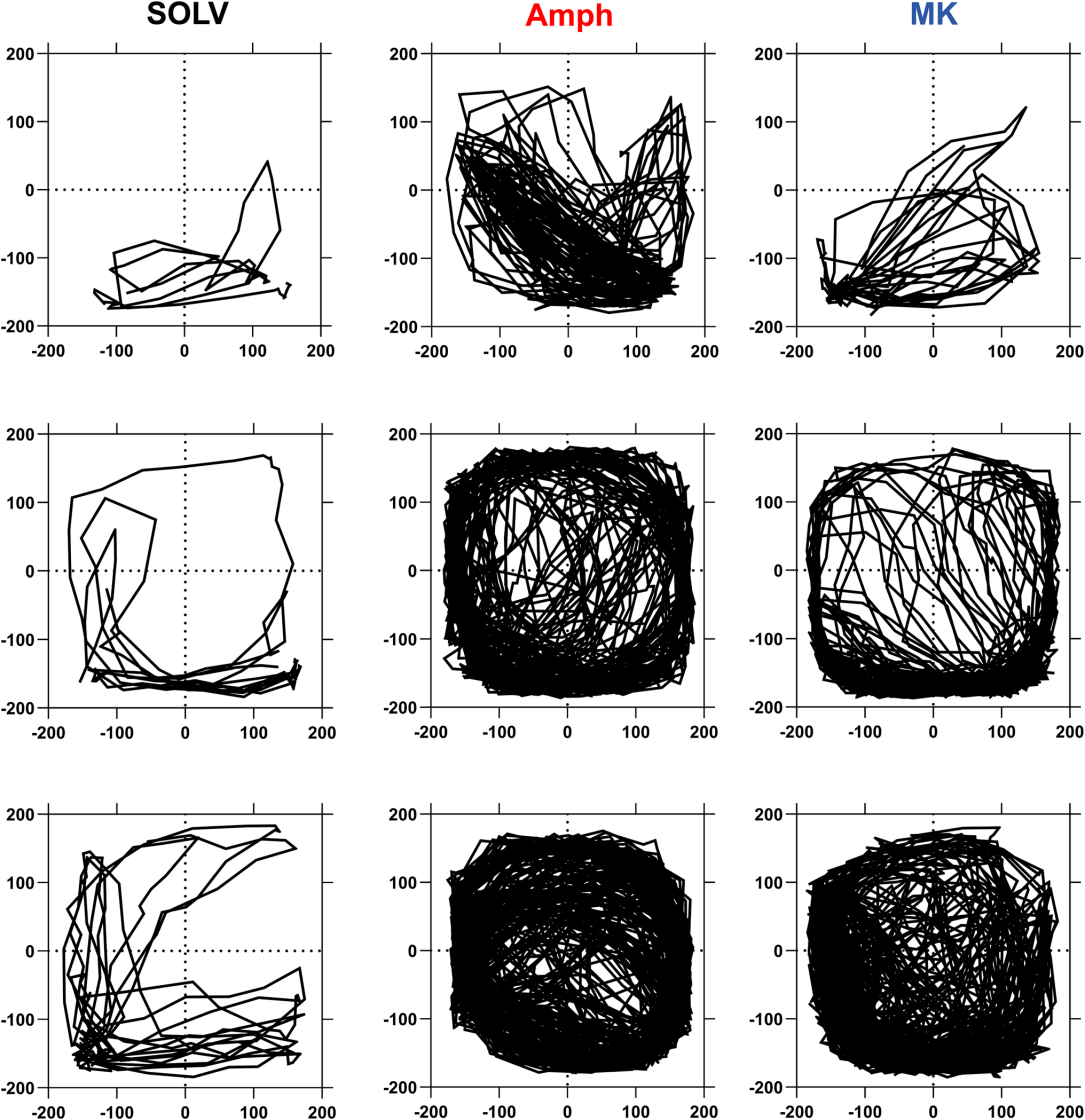
Representative trajectories (averaged for 60 min) of movements of animals treated with solvent (SOLV), amphetamine (Amph) and MK-801 (MK)

Immediately after amphetamine or MK-801 injections, rats were placed in the FPA. An animal was placed on a 40-cm × 40-cm plate covered by a Plexiglas enclosure (33 cm high) and put into a ventilated sound-attenuating chamber. Four force transducers located below the corners of the plate tracked the animal position on a Cartesian plane at each time point (Fig. 2). Data were collected during time units of 20.48 s (“frames”) with the sampling frequency of 50 points per second. The software calculated the total distance travelled in mm during three consecutive 60-frame series [three time periods of ca. 20 min each (20.48 min)] which was used as a measure of locomotor activity. Because vibration noise causes the measured position of the animal to fluctuate, this parameter could be artificially increased.

### Statistics

Statistical analyses were carried out using the software Statistica v.13.3 (TIBCO Software Inc., Tulsa, OK, USA). ANOVA for repeated measures was used followed by LSD post hoc test for individual comparisons.

## Results

### An influence of stimulation of adenosine A1 receptors on the amphetamine-induced increase in locomotor activity in rats

**Fig. 3:**
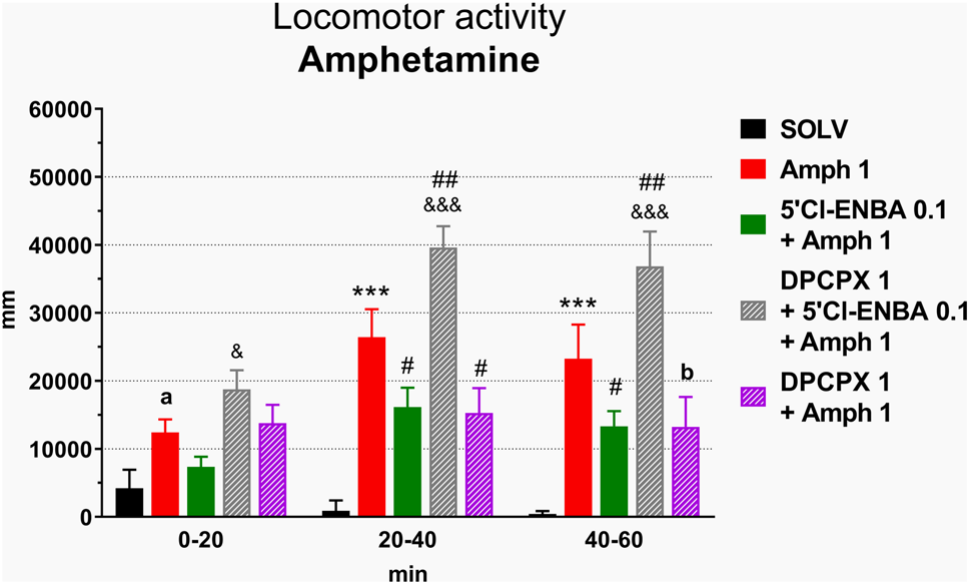
An influence of stimulation of adenosine A1 receptors on the locomotor activity increased by amphetamine in rats. Ordinate: the total distance travelled in mm, abscissa: the time after amphetamine injections. 5′Cl-ENBA 0.1, 5′-Cl-5′-deoxy-ENBA at the dose of 0.1 mg/kg; Amph 1, amphetamine at the dose 1 mg/kg; DPCPX 1, DPCPX at the dose of 1 mg/kg. SOLV, solvent. The number of rats in groups: SOLV, *n* = 8; Amph 1, *n* = 15; 5′Cl-ENBA 0.1 + Amph 1, *n* = 12; DPCPX 1 + 5′Cl-ENBA 0.1 + Amph 1, *n* = 9; DPCPX 1 + Amph 1, *n* = 7. Statistics: ANOVA for repeated measures: treatment effect (*F*[4,44]  = 10.6265, *p* = 0.0000), time effect (*F*[2,88] = 18.5794, *p* = 0.0000), treatment x time interaction (*F*[8,88] = 5.7804, *p* = 0.0000). LSD post hoc test: ***p < 0.001 vs. SOLV, ^a^*p* = 0.094 vs. SOLV, ^#^*p* < 0.05 vs. Amph, ^##^*p* < 0.01 vs. Amph, ^b^*p* = 0.058 vs. Amph, ^&&&^*p* < 0.001 vs. 5′Cl-ENBA + Amph

Amphetamine administered at a dose of 1 mg/kg sc increased the distance travelled by rats during the whole period (60 min) of recording (Figs. 2, 3). 5′-Cl-5′-deoxy-ENBA injected at a dose of 0.1 mg/kg ip inhibited the amphetamine-induced increase in locomotor activity by ~ 40%. A significant effect was observed between 20 and 60 min after amphetamine (50–90 min after 5′-Cl-5′-deoxy-ENBA). DPCPX (1 mg/kg ip) reversed the 5′-Cl-5′-deoxy-ENBA effect. However, DPCPX alone diminished the amphetamine-induced hyperactivity (20–60 min after amphetamine, 60–100 min after DPCPX) (Fig. 3).

### An influence of stimulation of adenosine A1 receptors on the MK-801-induced increase in locomotor activity in rats

**Fig. 4:**
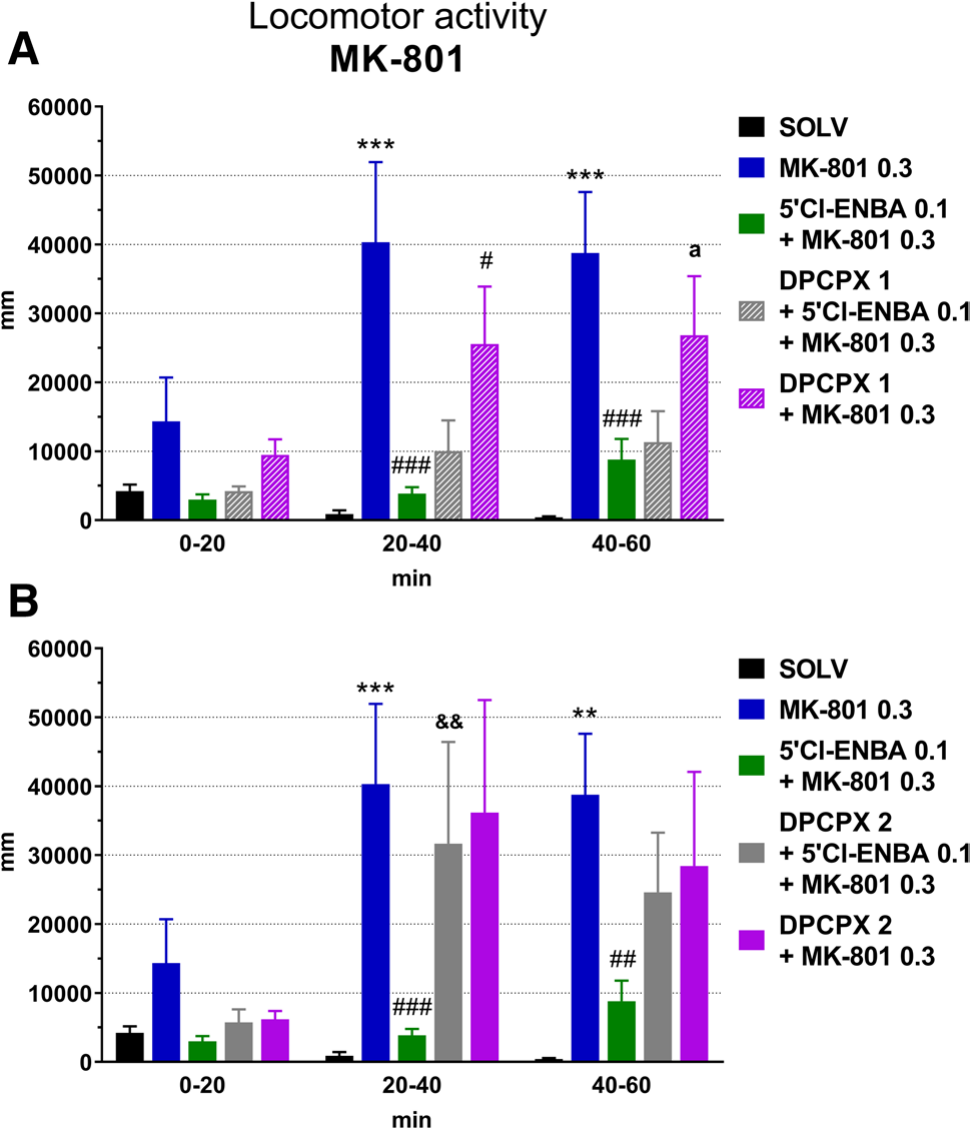
An influence of stimulation of adenosine A1 receptors on the locomotor activity increased by MK-801 in rats. DPCPX 1, DPCPX at the dose of 1 mg/kg; DPCPX 2, DPCPX at the dose of 2 mg/kg; MK 0.3, MK-801 at the dose of 0.3 mg/kg. The number of rats in groups: SOLV, *n* = 8; MK-801 0.3, *n* = 10; 5′Cl-ENBA 0.1 + MK 0.3, *n* = 12; DPCPX 1 + 5′Cl-ENBA 0.1 + MK 0.3, *n* = 9; DPCPX 2 + 5′Cl-ENBA 0.1 + MK 0.3, *n* = 11; DPCPX 1 + MK 0.3, *n* = 8; DPCPX 2 + MK 0.3, *n* = 6. Statistics for A: ANOVA for repeated measures: treatment effect (*F*[4,39] = 6.39543, *p* = 0.0005), time effect (*F*[2,78] = 17.23720, *p* = 0.0000), treatment × time interaction (*F*[8,78] = 4.74937, *p* = 0.0000). LSD post hoc test: ***p < 0.001 vs. SOLV, ^#^*p* < 0.05 vs. MK, ^###^*p* < 0.001 vs. MK, ^a^*p* = 0.098 vs. MK. Statistics for B: ANOVA for repeated measures: treatment effect (*F* [4,39] = 3.47610, *p* = 0.0160), time effect (*F* [2,78] = 13.8340, *p* = 0.0000), treatment × time interaction (*F* [8,78] = 2.6085, *p* = 0.0139). LSD post hoc test: **p < 0.01 vs. SOLV, ***p < 0.001 vs. SOLV, ^##^*p* < 0.01 vs. MK, ^###^*p* < 0.001 vs. MK, ^&&^*p* < 0.01 vs. 5′Cl-ENBA + MK. For further explanations, see Fig. 3

MK-801 injected at a dose of 0.3 mg/kg ip extended the distance travelled by rats (Figs. 2, 4). The significant effect was noted between 20 and 60 min after administration of this agent. 5′-Cl-5′-deoxy-ENBA (0.1 mg/kg ip) decreased the MK-801-induced locomotor activation (20–60 min after MK-801, 50–90 min after 5′-Cl-5′-deoxy-ENBA) by ~ 80% and this effect was inhibited by DPCPX given at a dose of 2 but not 1 mg/kg ip (Fig. 4a, b). DPCPX (1 mg/kg ip) alone diminished the MK-801-induced hyperlocomotion (20–60 min after MK-801, 60–100 min after DPCPX) (Fig. 4a). In contrast, the higher dose of this antagonist (2 mg/kg ip) did not influence the MK-801-induced hyperlocomotion (Fig. 4b).

## Discussion

The present study shows that 5′-Cl-5′-deoxy-ENBA, a potent and highly selective adenosine A_1_ receptor agonist reduced the amphetamine- and MK-801-induced hyperlocomotion of rats, which was reversed by the antagonist of these receptors DPCPX. Since hyperlocomotion induced by the above compounds is generally accepted to be a model of positive psychotic symptoms in humans [1], the present data suggest that stimulation of adenosine A_1_ receptors may produce antipsychotic effects.

The above conclusion corroborates our initial thesis about a potential antipsychotic effect of 5′-Cl-5′-deoxy-ENBA, which was based on the inhibition of the harmaline-induced hyperactivity [5]. In our previous study, we found a dose-dependent effect of 5′-Cl-5′-deoxy-ENBA (0.01–0.5 mg/kg) in the harmaline model [5]. On the basis of these experiments, we chose the dose of 0.1 mg/kg of this compound because of its marked effect on the harmaline-induced hyperactivity (ca. 40% reduction) and only slight inhibitory influence on spontaneous locomotor activity (ca. 20%) [5]. Similarly, the inhibitory effect of this dose on the amphetamine- and, especially, MK-801-induced hyperlocomotion observed in the present study was clearly stronger than that on spontaneous motility [5], which allows us to suggest real antipsychotic potential of 5′-Cl-5′-deoxy-ENBA. Moreover, a slight decrease in spontaneous locomotor activity observed by us earlier [5] is not predictive of a strong sedative effect in humans.

Hypermotility induced by amphetamines is generally accepted to result from an increased release of dopamine in the ventral striatum, i.e. nucleus accumbens [17]. On the other hand, activation of adenosine A_1_ receptors by systemic or local administration of their agonists has repeatedly been described to reduce spontaneous dopamine release in the striatum and in the shell subregion of the nucleus accumbens in vivo and in vitro [18–20], as well as the methamphetamine-enhanced release of this neurotransmitter in the striatum [21]. Accordingly, antagonists of these receptors induced an opposite effect, i.e. they increased dopamine release in the striatum [19] and nucleus accumbens shell [22–25]. In rodents and humans, adenosine A_1_ receptors (post- and pre-synaptic) are present in the striatum and nucleus accumbens [26, 27]. Some of them have been suggested to be localized on dopaminergic terminals in both these structures because their coding mRNA and/or protein expression was found in brain regions giving rise to mesolimbic and mesostriatal projections, i.e. in the ventral tegmental area [26, 27] and the substantia nigra pars compacta, respectively [27]. Moreover, an inhibitory effect of adenosine A_1_ receptors on adenylyl cyclase activity in the striatum was lost in rats lesioned with 6-hydroxydopamine [28]. Although adenosine A_1_ receptors located presynaptically on dopaminergic terminals may be involved in the decrease in exocytotic dopamine release shown by previous papers [18–20], their contribution to the inhibition of dopamine outflow [21] underlying hypermotility induced by amphetamines may be questioned because the latter is related to a reversal of dopamine transporter (DAT) function [29]. However, the stimulation of these receptors may inhibit dopamine synthesis in striatal varicosities and in this way it may lower its intraneuronal pool to be released by amphetamine. On the other hand, some lesion studies negated presynaptic localization of A_1_ adenosine receptors on dopaminergic terminals in the striatum [30]. Therefore, another indirect mechanism modulating dopamine efflux seems to be more plausible.

It is well known that stimulation of adenosine A_1_ receptors inhibits glutamate release in different brain structures, especially in the conditions when it is enhanced by, e.g. ischemia [31]. In line with this observation, an adenosine A_1_ receptor agonist decreased, whereas an antagonist increased the release of this neurotransmitter in the nucleus accumbens shell and striatum [22, 23, 25, 32]. Both these structures receive projections from the cerebral cortex: from the sensorimotor/motor frontal cortex (striatum) [33] and association medial prefrontal cortex (nucleus accumbens) [34]. Since adenosine A_1_ receptors are present on a majority of glutamatergic corticostriatal terminals where they form heteromers with A_2A_ receptors [30, 32], the above effects of the agonist and antagonist may result, at least partly, from their action on these presynaptic receptors.

A strong reciprocal dopaminergic–glutamatergic interaction exists in the nucleus accumbens. Accordingly, amphetamine has been found to increase the spontaneous release of glutamate in the nucleus accumbens [35], and vice versa dopamine release in this structure was enhanced by the stimulation of the cortico-accumbal projection and its terminals [34] via NMDA receptors [23], and the latter process involved dopamine reverse transport [29]. Therefore, it is supposed that the mechanism, which underlies the 5′-Cl-5′-deoxy-ENBA-induced reversal of the amphetamine-induced hyperlocomotion, might involve initially a decrease in glutamatergic transmission, which secondarily results in the diminished DAT-dependent dopamine release.

The mechanisms responsible for hyperlocomotion induced by MK-801, although not completely understood, seem to be different from those of amphetamine. Although uncompetitive NMDA receptor antagonists increase dopamine release in the nucleus accumbens and medial prefrontal cortex, these biochemical effects have been found to be temporally dissociated from hyperlocomotion [36], which persisted after a lesion of monoaminergic pathways [37]. Instead, a strong relationship between glutamatergic neurotransmission in the medial prefrontal cortex and this behaviour has been suggested [38–41]. In line with this concept, uncompetitive NMDA receptor antagonists (including MK-801) increase glutamate release in this structure which via AMPA/kainate receptors excites pyramidal neurons, i.e. increases their metabolic activity, early gene expression, and firing [39–44].

Adenosine A_1_ receptors have been found to modulate neuronal activity and glutamatergic transmission in the cerebral cortex. Immunoreactivity of these receptors was detected in almost all pyramidal neurons of II–VI cortical layers, with the strongest signal in the layer V [27], and their stimulation inhibited the synaptic transmission in these cells [45]. Moreover, an adenosine A_1_ receptor agonist suppressed glutamate and aspartate release in the prefrontal cortex [46]. Therefore, it seems that the decrease in MK-801-induced hyperlocomotion by 5′-Cl-5′-deoxy-ENBA, observed in the present study, may be related to its inhibitory influence on the prefrontal cortex. It is worth mentioning here that the above-described behavioural 5′-Cl-5′-deoxy-ENBA effect on the MK-801 model was much stronger than that in the amphetamine model and, therefore, twice as high dose of DPCPX was necessary to antagonize it. Such strong effect of 5′-Cl-5′-deoxy-ENBA may be explained by much higher density of adenosine A_1_ receptors in the cortex than in the basal ganglia (striatum, nucleus accumbens) in rats [26, 27].

Surprisingly, the present study shows that although DPCPX reversed the inhibitory effect of 5′-Cl-5′-deoxy-ENBA on the amphetamine- and MK-801-induced hyperlocomotion, this compound administered alone reduced both these behaviours. The mechanisms underlying this effect are unclear at present and may result from some unspecific properties of this antagonist. First, DPCPX has been reported to display both adenosine antagonist and adenosine agonist properties, the latter occurring at a site distal to cAMP, as shown in FRTL5 thyroid cell line [47]. Furthermore, while DPCPX is considered to be a potent and highly selective antagonist of A_1_ receptors in sub- to low nanomolar concentrations (Ki = 0.45–1.9 nM), it binds also to A_2A_ receptors in concentrations ca. 50–700 times higher (100–330 nM) [16, 48]. In our experiments, we observed the inhibitory effect on hyperlocomotion of a dose of 1 mg/kg, which is generally used to block adenosine A_1_ receptors in vivo [49]. However, DPCPX administered in mice at a dose as low as 0.25 mg/kg attains the level of ca. 340 nM in the brain, which is high enough to bind additionally to A_2A_ receptors [50]. In line with the putative dual action on A_1_ and A_2A_ receptors, DPCPX already at the dose of 0.1 mg/kg induced the effect characteristic of an antagonist of A_2A_ receptors, i.e. it reduced the ischemia-evoked aspartate and glutamate release in the cerebral cortex in rats [31]. In contrast to the cerebral cortex, the A_2A_ component of DPCPX does not seem to influence striatal neurotransmission. Although antagonists of A_2A_ receptors have been found to inhibit spontaneous or stimulated dopamine and glutamate release in the striatum and nucleus accumbens shell [24, 34, 51, 52], especially in conditions of concomitant blockade of A_1_ adenosine receptors [19], the intrastriatal administration of DPCPX did not reduce the methamphetamine-enhanced extracellular dopamine level [21]. Moreover, it seems controversial, whether A_2A_ component of DPCPX may contribute to its effect on the amphetamine- and MK-801-induced hyperlocomotion. Although, knockout of A_2A_ receptor in mice has been found by Moscoso-Castro and co-workers to decrease both spontaneous locomotor activity and that increased by amphetamine or MK-801 [53], other authors have shown either a lack of effect or an opposite effect of antagonists of these receptors [1, 2].

Stimulation of adenosine A_1_ receptors has already been suggested to be related to the potential antipsychotic action [1]; however, agonists of these receptors slow down the heart rate and decrease systolic blood pressure in animals which may limit their use in humans [7, 10]. 5′-Cl-5′-deoxy-ENBA seems to be devoid of systemic side effects of other A_1_ agonists because in centrally effective doses (up to 0.5 mg/kg) in mice it neither induced cardiovascular effects nor disturbed motor coordination [12]. The reason of the lack of cardiovascular effect of this compound is unknown. It may stem from its ability to target mainly cells highly expressing adenosine A_1_ receptors, which was suggested by Luongo and co-workers [12]. In fact, density of these receptors in the heart is low in comparison to the brain [16]. However, another explanation may also be proposed. A recent study in knockout mice has shown that the activation of adenosine A_3_ receptors contributes to cardio-depressant effects of an adenosine analogue [54], and compounds expected to act selectively on adenosine A_1_ receptors, at doses which strongly reduced heart rate [7, 54], influenced also adenosine A_3_ receptors [14, 54]. 5′-Cl-5′-deoxy-ENBA is the most selective agonist of adenosine A_1_ receptors currently known [11, 14] which might explain the lack of its peripheral actions [12]. This suggestion is supported by the recent finding that, at the dose used in the present study, it did not induce peripheral adenosine A_3_ receptor-dependent hypothermia in mice [14].

Summing up, the present study suggests potential antipsychotic action of 5′-Cl-5′-deoxy-ENBA, a potent and selective adenosine A_1_ receptor agonist, which seems to be devoid of serious peripheral side effects.
